# Potential of blockchain approach on development and security of microbial databases

**DOI:** 10.1186/s12575-020-00139-z

**Published:** 2021-02-01

**Authors:** Fatemeh Mohammadipanah, Hedieh Sajedi

**Affiliations:** 1 Pharmaceutical Biotechnology Lab, Department of Microbiology, School of Biology and Center of Excellence in Phylogeny of Living Organisms, College of Science, University of Tehran, Tehran, 14155-6455 Iran; 2 Department of Computer Science, School of Mathematics, Statistics and Computer Science, College of Science, University of Tehran, Tehran, 14155-6455 Iran

**Keywords:** Microbial databases, Blockchain technology, Bacterial supply chain, Culture collections, Global data exchange, Biobanking

## Abstract

**Abstract:**

Approaches developed based on the blockchain concept can provides a framework for the realization of open science. The traditional centralized way of data collection and curation is a labor-intensive work that is often not updated. The fundamental contribution of developing blockchain format of microbial databases includes: 1. Scavenging the sparse data from different strain database; 2. Tracing a specific thread of access for the purpose of evaluation or even the forensic; 3. Mapping the microbial species diversity; 4. Enrichment of the taxonomic database with the biotechnological applications of the strains and 5. Data sharing with the transparent way of precedent recognition. The plausible applications of constructing microbial databases using blockchain technology is proposed in this paper. Nevertheless, the current challenges and constraints in the development of microbial databases using the blockchain module are discussed in this paper.

**Graphical Abstract:**

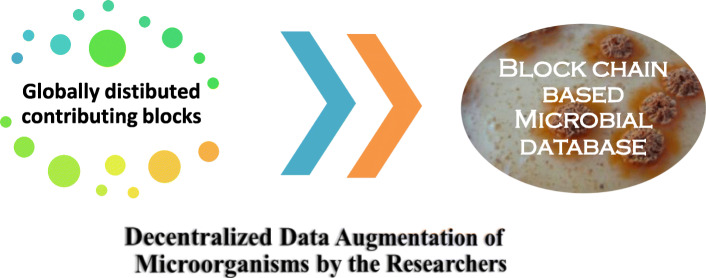

## Highlights

- Implementation of blockchain for building and managing of the microbial databases is proposed
- Continuous multicenter teledata deposition of microbial strains can be provided by the development of some platforms based on the blockchain concept
- Tracing a specific thread of access for the purpose of evaluation or even the forensic will be possible in blockchain based database of bacteria
- Mapping the microbial species diversity will become possible by using this concurrent data filling from different blocks
- Enrichment of the taxonomic database with the biotechnological application of the strains will be globally accelerated by this instant cooperative data deposition in blockchain-based architecture

## Background

### The Mathematical Concept of Blockchain

Blockchain is a database with the capability of duplicating, sharing, and synchronizing data distributed through different physical places (i.e., various organizations, countries, etc.) [1]. Nowadays, there is a tendency to ignite different non-financial fields by using the newly emerging computational tools of blockchain. Recently, blockchain has been gradually used to handle multidimensional tasks in health care, assets, networking, electronic voting, etc. Nevertheless, factors such as block production rate, transaction speed, and block size, play a decisive role in the overall performance of the blockchain-based solutions [2].

A blockchain is a data infrastructure that retains and shares all transactions made since its inception. It is mainly a fragmented distributed database that keeps a list of gradually growing data records protected from manipulation and unauthorized access. In a blockchain, a user can connect to the network, create new blocks, submit new transactions, and confirm them. An encryption hash is assigned to each block (i.e., a block fingerprint) that is valid until the data in the block changes. If there are any changes in the block, the encryption hash changes quickly, causing changes in the data that may be due to illegal activity. Thus, due to its solid foundations in cryptography, blockchain is gradually being employed to reduce unauthorized transactions in different fields [2].

The paper starts with the operational prerequisites of blockchain implementation, and the second section mentions the current non-financial fields of blockchain adaptation. The third section reveals the fundamental concepts of applying blockchain technology in the creation and management of microbial databases. The fourth section elaborates on the supposed properties of the future microbial databases that will develop based on the blockchain platform. Subsequently, some added value of using blockchain in designing the microbial database is proposed. In the last section, some challenges and limitations in implementing the blockchain for the purpose of building microorganism databases are discussed.

#### Consensus Algorithms

The consensus layer is one of the essential layers in any blockchain-based system. This layer is created to maintain the reliability of the network, assuming that there are untrusted users.

Consensus algorithms are responsible for achieving an agreement of distributed systems on a certain amount of data. The role of consensus algorithms is to provide reliability in a network that could have unreliable nodes. Reliability is vital in distributed systems and databases.

There are several consensus algorithms, and each one has its advantages and weaknesses. In public blockchains, usually, Proof of Work (PoW) or its extensions are used as consensus algorithms [1]. PoW refers to the calculation of the hash value of a block with the required number of main zeros by changing a random number. This process is identified as mining, and it is a process with high processing or energy requirements. When a miner finds a valid node, broadcasts it to other nodes for verification.

Consensus algorithms necessarily assume that some processes and systems are not available and that some communications will be lost. Therefore, consensus algorithms must be designed to tolerate error.

#### Blockchain-Based Management Style

Blockchain can help different institutions managing databases by increasing the speed of operations, spending less, reducing individual errors, and improving the level of information security (Table 1).

**Table 1:** Blockchain Technology

This is an open distributed ledger consist of records of data across many computers that are resistant to modifications. The networks of records (blocks) are linked using cryptography and each block contains a cryptographic hash and timestamp that link it to other blocks. A blockcain database is usually managed autonomousy using a peer-to-peer network collectively adhering to a protocol for inter-node interactions or confirming the creation of new blocks. Therefore, features of this system that its block content cannot be modified retrospectively, without the alterations of all subsequent blocks. This allows the tracking the thread of data deposition and robust security. The database building with the blockchain model doesn’t need constant curation and therefore participants can audit the data deposition and exchanges. By using this technology of data deposit and sharing system, in addition to the decentralization that is favored in the globalization of the science, instant cooperative data deposition becomes possible.	

The key specification of blockchain databases is that there is no central supervisor or centralized data storage mechanism. Instead, consensus algorithms manage the decentralized network [1] and no organizational authorities are required.

#### Models of Security Assurance and Legitimacy Issues

Blockchain technology combines concepts such as peer-to-peer protocols, hashing algorithms, primary encryption, public-key encryption, and consensus algorithms. A blockchain is based on a decentralized network which its main task is protecting stored lists of records against tampering.

The blocks in the blockchain are interconnected, forming a chain of blocks. The first block is called the “origin” block [1]. All nodes are connected to a flat topology without a central reference or the main server. This structure of the peer-to-peer network makes it completely decentralized [3]. A ledger is kept in the blockchain, where all committed transactions are stored in a list of blocks. The chain develops as the new blocks are joined gradually.

In a peer-to-peer network, a consensus mechanism is used to ensure that this block is valid before it is recorded in the ledger. After registering the block in the ledger, the entire network gets a copy of the updated ledger. Participants in the blockchain network are allowed to view the digital ledger, which is shared securely through the distributed computer network. All peer-to-peer network nodes access have peer-to-peer data and create an independent network to generate and share data between these nodes. Afterward, the blockchain will be the only basis of its validation.

Finally, knowing that in the court in China, it is possible to use the Chinese blockchain to validate documents in legal disputes [4], will ensure the security of the blockchain.

## MAINTEXT

### Current Non-financial Applications of Blockchain Technology

The blockchain technology applied for the cryptocurrency is now being extended to other non-financial fields of technologies. This approach is penetrating to other disciplines that can be revolutionary for the usual way of data sharing the world is used to that.

Some unified aspects like an open way of data exchange, prevention of monopolies supervision, the secure validation processes of the transaction, etc. has made this technology attractive for adopting in the different non-financial sector including the academia and biotechnology sectors.

**Fig. 1:**
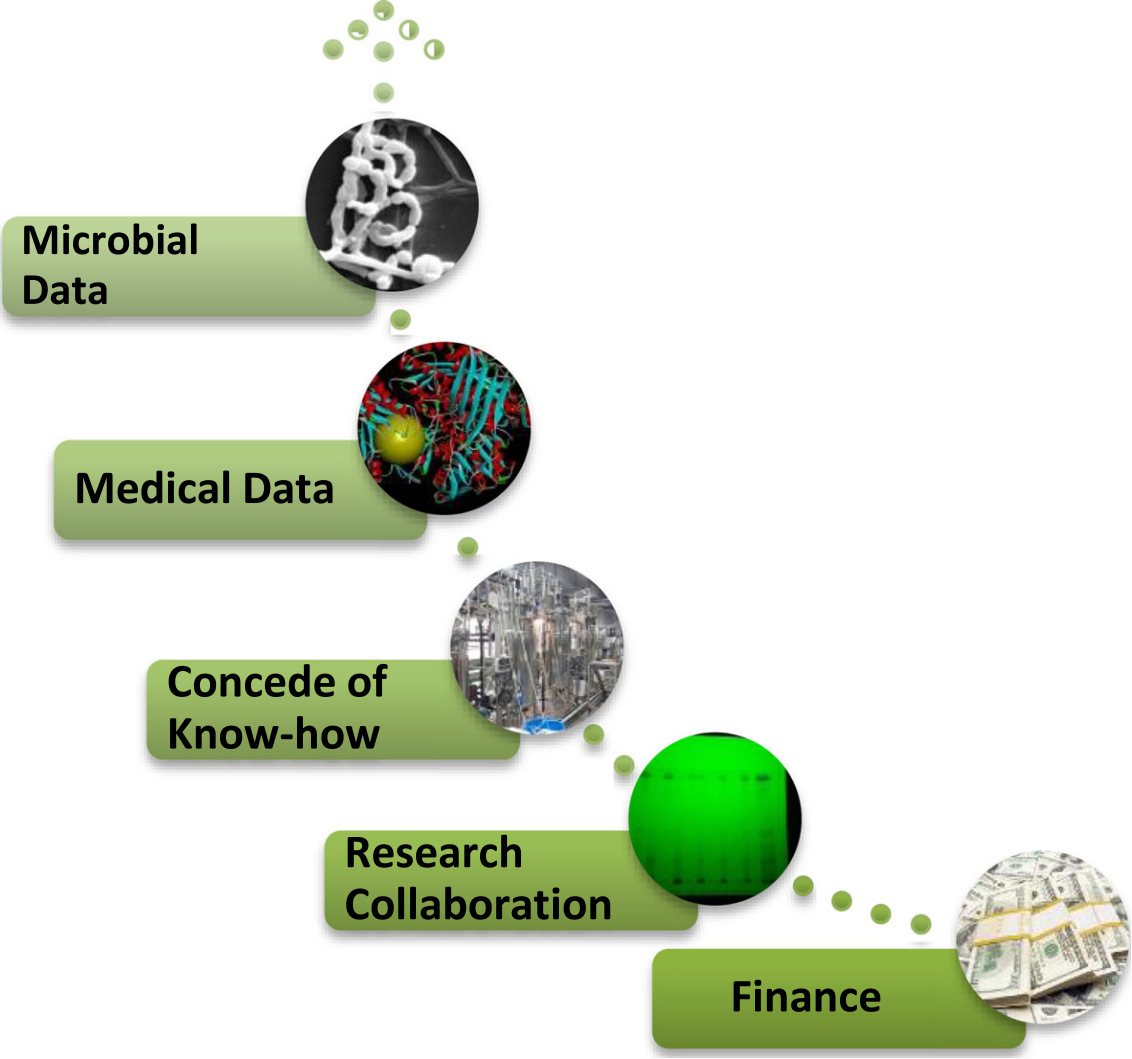
Overall evolutionary trend of applying the blockchain technology in science

Based on a recent survey the majority of the journal publications describing the new application of blockchain technology has been in the field of Internet of Things (IoT), energy, healthcare, finance, resource management, government, exchange, rights management, privacy, supply chain, etc. [5]. Blockchain technology is employed mainly for sharing medical data in the last few years, and prototype tools for this purpose and companies to establish this infrastructure has emerged [6]. For instance, clinical trial data validation of a breast cancer drug was successfully conducted using a blockchain data system to verify the adherence to the protocol from distributed cloud server services [7]. The main disciplines in science that blockchain tools are developed so far for them is illustrated in Fig. 1.

#### Decentralized Network

Decentralization can remove the large flows of traffic to one node (i.e., server), provides the strength to prevent delays and tolerating the failure of the server.

#### Auditability

In blockchains, the entry, retrieval, and exchanges of data are validated and recorded by a timestamp. This property provides the users to trace the former records directly by access to any node of the network. In regular centralized databases, a person or group owns it and can make changes to it or destroy information.

#### Transparency

All the transactions in public databases are transparent and tractable. The records of the activities can be made public so that they will be visible to all participants. Although, the level of participant’s access can also be organized as needed.

#### Veracity

Blockchain ensures the accuracy of the stored records because the same version of the historical ledger records is replicated and stored in the network nodes. In addition, each case is approved by consensus. If fake inputs are entered, they will be detected and eliminated based on the consensus algorithm.

#### Scalability

Scalability is the ability to afford accommodation for the volume of work and provide storage space by increasing the number of strains or objects.

### Potential of Blockchain in Management of Microbial Databases

All pure and applied microbiology research area is impacted by the genetic and phenotype stability of the microorganisms being used as in the experiments either as the object of the study or the reference strains. This stability and reproducibility are needed to sustain productivity in biotech and pharmaceutical companies [8]. This genetic stability as a constant concern of the academia and industry is conserved by the prevention of microorganisms in Culture Collections (CC).

CCs are devoted institutes with public service over 100 years to provide authentic, safe, preserved biological material with their associated data to support the long-term supply for reproducible research/production. Biological Resource Centers (BRC) are more comprehensive culture collections focused on supplying high-quality resources (not limited to reproducible cells) into biotechnology research and development.

Reproducibility of the biotechnological exploitation of microbial sources is warranted by the proper long-term preservation of these cells in CCs or BRCs.

Using the blockchain approach in the creation or expansion of a microbial database will remove the hurdles associated with the centralized data entry of a vast number of samples.

Most of the comprehensive microbial databases are created by the CCs, BRCs, or their consortia, namely WFCC (Table 2). There are some other microbial databases which have been constructed by other organization rather than collections or their consortia. For insatance, the construction of the comprehensive database of BacDive demanded manual insertion of strain characteristics from 782 papers in the International Journal of Systematic and Evolutionary Microbiology [9]. Although, such species descriptive papers only provide data of type strains and not all the species and strains.

**Table 2:** Current most comprehensive microbial phenotypic databases

Global Catalogue of Microorganisms (GCM): an information-sharing system between culture collections providing the data sharing possibility between the collections and presents a uniform interface for the users	
World Data Center for microorganisms (WDCM): a data center for networking the microbial resource center and an information database for the strain customers	
Bacterial Diversity Metadatabase (BacDive): contains data on the morphology, physiology, taxonomy, cultivation, isolation, and genomic data of 63,669 bacterial, including 12,715 type strains	
Culture Collection Information Worldwide (CCINFO): a database consists of a list of strains from 791 registered culture collections of 78 countries	

There is still not a prototype of a blockchain-based microbial database available to the public. There are two main plausible ways by which microbial databases can benefit from blockchain technology:

1. Data enrichment of the already existing microbial databases.
2. Creation of the Strain Database that Will Be Filled from Zero from Numerous Spread Blocks from all over the World

#### The Infrastructure of a Blockchain-Based Microbial Database

Microbial databases can leverage blockchain capabilities to achieve integrity, and non-repudiation which are essential for the microbial reference databases.

**Fig. 2:**
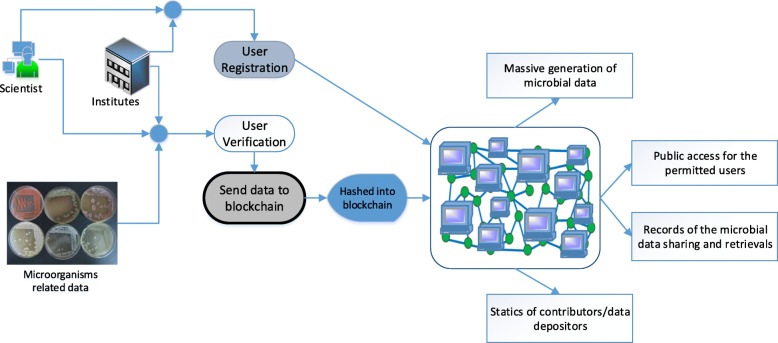
A blockchain-based microbial database

In this paper, we suggest implementing a public blockchain infrastructure while minimizing the corresponding monetary cost for building a microbial database. We propose a blockchain-based microbial data collection system demonstrated in Fig. 2. The blockchain mode can assist in building a pool of biological data by creating a unique address for labs, institutes, etc. which can be used to represent the local physical information. In this scheme, the microbial data, including images, physiological traits, sequences, chemotaxonomical data, etc., scientist identity, along with their scientific affiliations can be recorded. The sequence of 16S RNA or genome must be defined as the mandatory data required for strain data deposition in each block while some other characteristics such as biochemical or morphological data could be assigned as the optional fields which are not essentially required for deposition in a block while remain amenable to completion by the time when additional data will be available for the strains.

The overall features of blockchain for microbial databases developed based on the blockchain technology can be considered as transparancy, auditability, veracity and scalibility.

Scalability of the microbial databases is of importance as the predicted species of just bacteria surpass one million species while there are multiple of this value of different strains at the lower taxonomic level of species ie. strains. Around 35.5 million physiological functions in bacteria and 3.2 million in fungi are predicted out of which only 0.02 and 0.14% are discovered until now, respectively. Therefore, there is still a lot of microbial physiology waiting to be discovered and biobanked [10].

Below are some supposed features that the blockchain-based databases of microorganisms can possess and the privileges that can be obtained by implementing this technology in the management of the microbial data for CC, BRCs, or research or industry institutes and individuals are summarized in the below subsections.

#### Metadata of Strain Isolation

Modifications in original data inserted by the institute managing the database are prohibited in all current databases of microorganisms.

When the user with approved identity has the permission to add more characteristic information of the strains, the metadata of the strain will be more enriched as the complementary data on each strain will be discovered by the time during different research projects at various parts of the world.

In addition, the scattered reports on webpage, reports, papers, etc. can also be directly added to the database by the scientists from different block points. Therefore, the huge dispersed data can be deposited in an integrated database either public, partially encrypted to the defined users, or fully encrypted by cryptographically signing the data.

By establishing such integrated databases, the same conditions for global development, and the progress of technologies related to the microbial resources will be provided to all scientists.

Moreover, the meta-analysis of such collected big data can itself lead to an additional interpretation that can be beneficial to reveal the usage pattern of the strains at a global level.

#### Data Sharing

The distributed databases of the culture collections from all over the world (966 registered collection till November 2020) can be synchronized that has always been required and dreamed by the microbiologist to have a single comprehensive data search from some comprehensive microbial databases such as the databases indicated in Table 2.

Such a disruptive way of data sharing can affect the rate of the species data exchange between academia and industry. In accordance, the interaction will increase the advancement in microbial biotechnology. The intrinsic trait of transparency in the map of the exchanged data, itself provides an accurate conclusion on the existing flow of the knowledge in this field of science between participants and counterparts.

In sharing data under a blockchain format, not only the data deposition, and exchange can be monitored, even the access, and view and of the sensitive data can also be controlled.

The blockchain-based systems can also be set in such a way to allow only part of these biotechnological data at a defined charge to be accessible for profit-making users such as private biotechnological or pharmaceutical companies.

#### Project Data Management

Among all, project data management will ultimately be affected sooner or more extensively by blockchain technology. Encrypted information in the blockchain-structured databases can be shared between researchers or institutes without limitations or interventions. The shared network is scalable and can proceed without the need for any verification process dictating from a third party.

Although all data entry, retrieval, or exchanges are immutable, certain credentials can be adjusted to modulate the full access to the content of each block. Therefore, data will be unhackablly maintained online.

For instance, the privileged access to some part of the database can be stratified between different groups that are involved in the microbiology projects.

The blockchain-based platforms can have the possibility in which researchers and industry can exchange early outcomes of the projects. These disperse database connection support uploading both confidential and public results of the research in the blocks. By the verification of the data depositor in each block, the biotechnological potential of the microorganism will be date-stamped for that researcher.

The associated data such as raw lab measurements, electron micrographs, spectra, related SOPs, graphs, tables, genomic sequences, etc. can also be merged to the strain deposited data in each block for accurate and effective data recording and sharing. The project collaborators involved in a microbial project from all over the world can add even further online amendment or create sub-block with appending data to the already existing data in each block.

#### Security

Among the core features of the culture collections is having a mechanism to secure their assets, the physical security of holdings, and the privacy of the associated data of the strains.

The mechanism employed in the blockchain tools, is to create an ultra-secure system for the storage of scattered data. The risk associated with the centralized system is resolved by cybersecurity associated with the distributed blocks. The digital forensics, in case of rare occurrence is repudiation free due to the transparency of the committed IP.

The security of the strain supply chain is the concern of the national and international bodies which is often resolved by limiting the distribution of the strain with BSLs higher than BSL2. This is not sufficient as bacteria from BSL 1, and 2 also have the dual-use potential or can be manipulated in a lab equipped with even minimum or medium technologies.

Moreover, computing servers used by microbial databases can be hijacked for other purposes, even cryptocurrency mining or other malicious purposes [11].

Each country has its own national policies and legislation on international exploitation of its genetic resources including the microorganisms. The adherence of the research entities to these regulations n acquisition, conservation, utilization and data sharing of microbial resources currently cannot be properly audited. However, monitoring the deviation from these national regulations is currently challenging and not properly possible. By employing the blockchain approach, due to their intrinsic trait of immutability, adhering to these regulations can be monitored by the authorities with a higher accuracy. In fact, a new type of auditory at the national/global level can track the microbial data deposit, access, and share across the national boundaries.

### Challenges in Applying Blockchain to Microbial Databases

Among the challenges in implementing the blockchain technology for building the microbial databases is that the modification of content in each block is not quite real-time and needs the permission of more than 50% of all segmented nodes. This demands the approval of more than half of participants that inevitably make any changes to each particular block automatically by kind of verification process. Although, this inevitable authentication process will bring along the integrity, clarity, and creditability in the data deposition. Additionally, the possibility of data breach when needed is estimated to rather zero in case of applying a blockchain approach.

Another concern is that although blockchain technology seems to be an emerging novel type of database curation in all disciplines as well as Microbiology, decentralized blockchain technology delete the necessity of trusted intermediaries for data exchange of the strains.

The third main concern is that the distribution of the strains or data by blockchain systems may not comply with international conventions and regulations like Nagoya Protocol. The entities that are involved in data sharing will not have to follow the classic way of hierarchical permission bureaucracies and is in a manner of consensus-driven that, in some cases, may violate the national or international regulations.

Some other limitation of recruiting blockchain-based database management is privacy concerns, off-chain transactions, loss of discretion and arbitration challenges and distrust of the technology due to lack of adoption [12].

Some other constraints of applying the blockchain format database building for microbial data can be summarized as:

- Incompatibility and variations in the format and unit of the deposited data
- The entry of the invalidated and tentative data of the strains
- Data removal by the blocks
- Manipulation of the data at the level of each block by the co-users

## Conclusion

### Concluding Remarks

Although blockchain emerged as a kind of Fintech, it is penetrating many other fields to deliver a similar service to what it has in the financial transaction system.

Here we discussed the data curation practices in BRCs or CCs that can be subjected to blockchain-based architecture. The labor of data entry can be mitigated by cooperative data insertion from different users. The origin of the inserted information will be clear and recognizable. At the same time, the direct in situ deposition of the strain data act as repositories for the protection of intellectual property. As the accuracy of all the predictions based on machine learning algorithms depends on the size of the microbial database [13], attaining a more comprehensive database will lead to a more precise computational analysis of the microbial debases using machine learning tools.

In the case of the incentivization of key stakeholders such as WFCC and ECCO, these tools can be developed to initiate the decentralized and synchronized type of data sharing. The massive burden of data entry and curation of the culture collections can turn into a lighter task for these service centers if the blockchain-based microbial database comes into existence.

Furthermore, since the billions of the investment required for strain exploitation, phenotypic, and genotypic analysis, letting the data integration from distributed blocks dramatically can decrease the cost of adding value to the strain data repositories.

Nonetheless, unique policies need to be decided based on a consensus of the majorities, to legislate the formation of such scalable specified databases for the strain characteristics.

Some guidelines for maintaining the biosecurity is formally announced for the BRCs [14], however still not sufficiently applicable in all culture collections. With the advance of synthetic biology and the availability of whole genomes data, there has been increasing concern on open access to such sequences in the microbial or genomic databases. By using a database constructed based on the blockchain algorithms, the access and users of pathogenic microorganisms data can be identified. Additionally, in the case of emerging new pathogenic strains or viruses, its associated data can be shared globally without national restrictions through recording in the blocks by individual scientists early enough to diminish the rate of dissemination and morbidities such as what happened in the recent COVID-19 pandemic.

### Future Perspective

The reliability and real-time update of the data are an essential part of the future advance in all parts of science and technology.

As there has been introducing some prototype tools designed based on the blockchain technology for medical data sharing, we expect some data-sharing tools to be developed between culture collections across the world based on the blockchain concept. Continuous multicenter teledata deposition of microbial strains can be provided by the development of some platforms based on the blockchain concept.

Using the decentralized validation of the strains exchanges, such functions can be accelerated and also be traced flawlessly in case of any illegal exchange. This type of data sharing can dramatically increase the amount of associated data for each stain and overall increase the transparency of the strain movement in the academy and industry. Following the implementation of blockchain technology for constructing the microorganism database and emerging some prototype, some real constraints and privileges of this type of database for the microbiology field building will be revealed in practice.

The improvements in strategies of cyberbiosecurity following the current development in genome data generation of pathogenic microorganisms sound more critical in the future (Table 3).

**Table 3:** Forthcoming possibilities

• What extra features will be possible and can be expected by applying blockchain technology for database construction of the microorganism?	
• Can we visualize the network of strain data exchanges and data deposit using blockchain-based developed databases?	
• Access to the pathogenic microorganisms data can be identified for the for biosecurity purposes?	
• Direct deposition of the strain data in the blockchain based databases will act as repositories for the protection of intellectual property?	
• Can strains exchanges be approved and traced flawlessly in case of any illegal exchange?	
• Map of the global biotechnological exploitation data can be readily constructed for interpretations of the trend of the works?	
• Establishing a clear legal frame on use and the provenance of the strain supply/exchange will be possible for the blockchain based microbial databases?	

Some speculations of the probable applications of using blockchain for building the microbial data are presented as forthcoming possibilities in Table 3. Nevertheless, one of the critical challenges in developing the blockchain tools is establishing a clear legal frame on use and the provenance of the strain supply/exchange that is favored to be designed before these blockchain tools leverage to the practice.

## Data Availability

There is no associated experimental data for this review to be provided.

## Abbreviations

CC: Culture Collections
BRC: Biological Resource Centers
WFCC: World Federation for Culture Collections
ECCO: European Culture Collections
SOP: Standard Operating Procedure
COVID-19: Coronavirus Disease of 2019
Fintech: Finantial Technology
PoW: Proof of Work
IOT: Intrnet of Things
IP: Intelectul Property
